# Crosstalk between gut microbiota and RNA N6-methyladenosine modification in diabetic retinopathy

**DOI:** 10.3389/fcell.2026.1841882

**Published:** 2026-08-04

**Authors:** Junyan Hu, Shuai Dong, Ziyi Yao, Ziqing Gao, Shengqun Jiang

**Affiliations:** 1 Department of Ophthalmology, First Affiliated Hospital of Bengbu Medical University, Bengbu City, Anhui, China; 2 Department of Ophthalmology, Dalian Third People’s Hospital Affiliated to Dalian University of Technology, Dalian City, Liaoning, China

**Keywords:** diabetic retinopathy, gut microbiota, gut-retina axis, metabolic memory, RNA N6-methyladenosine

## Abstract

Diabetic retinopathy (DR) is a leading cause of blindness in working-age adults, with a pathogenesis that extends far beyond chronic hyperglycemia. This narrative review synthesizes current evidence to construct a comprehensive model of DR that integrates metabolic memory, epigenetic regulation, and systemic factors such as the gut-retina axis. Specifically, this review focuses on the bidirectional crosstalk between gut microbiota dysbiosis and host RNA N6-methyladenosine (m6A) modification as the central mechanism linking these factors. We examine how gut microbiota dysbiosis contributes to the initiation and progression of DR by influencing the host epigenetic landscape, particularly the dynamic RNA N6-methyladenosine (m6A) modification. Hyperglycemia and microbial dysbiosis collectively drive the dysregulation of m6A “writers” (e.g., METTL3/14), “erasers” (e.g., FTO, ALKBH5), and “readers” (e.g., YTHDF family), leading to stable alterations in the expression of genes involved in inflammation, oxidative stress, angiogenesis, and neurodegeneration, thereby establishing “metabolic memory.” The gut microbiota and its metabolites (including short-chain fatty acids, secondary bile acids, trimethylamine N-oxide, and tryptophan derivatives) not only modulate host m6A modification but are also themselves influenced by the host m6A machinery, forming a bidirectional “microbiota-m6A” regulatory axis. A deep understanding of this interaction network not only offers a new perspective on the complex pathological mechanisms underlying DR but also lays a theoretical foundation for developing novel microbiome- and epitranscriptome-based biomarkers and therapeutic strategies, such as probiotics, prebiotics, and small-molecule drugs targeting m6A enzymes.

## 1 Introduction

Traditional pathological models of diabetic retinopathy (DR) have focused on the classical biochemical pathways directly activated by hyperglycemia, such as the polyol, hexosamine, protein kinase C (PKC), and advanced glycation end-product (AGE) pathways, which ultimately converge on oxidative stress and chronic low-grade inflammation (Hussain et al., 2023; Mahajan et al., 2019). However, the clinically observed phenomenon of “metabolic memory”—where the risk of DR persists even after subsequent glycemic control is achieved—suggests the existence of more durable molecular mechanisms (Khan et al., 2026). Epigenetic regulation, particularly DNA methylation, histone modifications, and non-coding RNAs, is considered a key mediator of this “metabolic memory” (Khan et al., 2026; Nolan et al., 2011). This review specifically focuses on the bidirectional crosstalk between the gut microbiota and RNA N6-methyladenosine (m6A) modifications in diabetic retinopathy.

In recent years, the Frontier of epigenetic research has expanded into the field of epitranscriptomics, where N6-methyladenosine (m6A), the most abundant and dynamic chemical modification in eukaryotic mRNA, plays a central role in cellular stress responses and homeostasis by regulating RNA splicing, nuclear export, stability, translation, and degradation (Wang et al., 2022; Zhuo et al., 2022). Concurrently, the gut microbiota, acting as a “superorganism,” has been closely linked to diabetes and its various complications through compositional and functional disturbances (i.e., dysbiosis) (Maruvada et al., 2017; Mokkala et al., 2021). The concept of the “gut-retina axis” connecting the intestine to the retina has been clearly proposed, with dysbiosis driving systemic inflammation—and consequently affecting retinal health—by disrupting the intestinal barrier, translocating microbial products (e.g., lipopolysaccharide), and altering metabolite profiles (e.g., short-chain fatty acids, trimethylamine N-oxide) (Huang et al., 2021; Li et al., 2023; Lin et al., 2025).

Emerging evidence indicates a profound bidirectional interaction between the gut microbiota and host m6A modifications (Wang H. et al., 2025; Zhang et al., 2025a; Liu Y. et al., 2025). On one hand, microbial metabolites can directly or indirectly act as substrates or regulators, influencing m6A modification patterns in host cells. On the other hand, the host m6A machinery can shape the intestinal environment and microbial composition. In the context of DR, hyperglycemia and gut dysbiosis may synergistically disrupt m6A homeostasis in the retina and systemically, thereby solidifying pathological gene expression programs. This review aims to systematically elucidate the interaction between the gut microbiota and m6A modifications in DR, integrating the concepts of metabolic memory, epigenetics, and systemic factors, while also exploring their potential for translational applications.

Recent clinical studies, particularly from Indian and Chinese cohorts, have begun to define DR-specific gut microbial signatures that distinguish DR from other diabetic complications (Huang et al., 2021; Khan et al., 2024). These studies reveal that DR patients exhibit a distinct microbial profile characterized by pronounced depletion of butyrate-producing genera and enrichment of pro-inflammatory taxa, providing a microbial basis for disease-specific therapeutic targeting.

## 2 Hyperglycemia-driven classical pathways and metabolic memory

### 2.1 Classical biochemical pathways: from hyperglycemia to retinal damage

**FIGURE 1:**
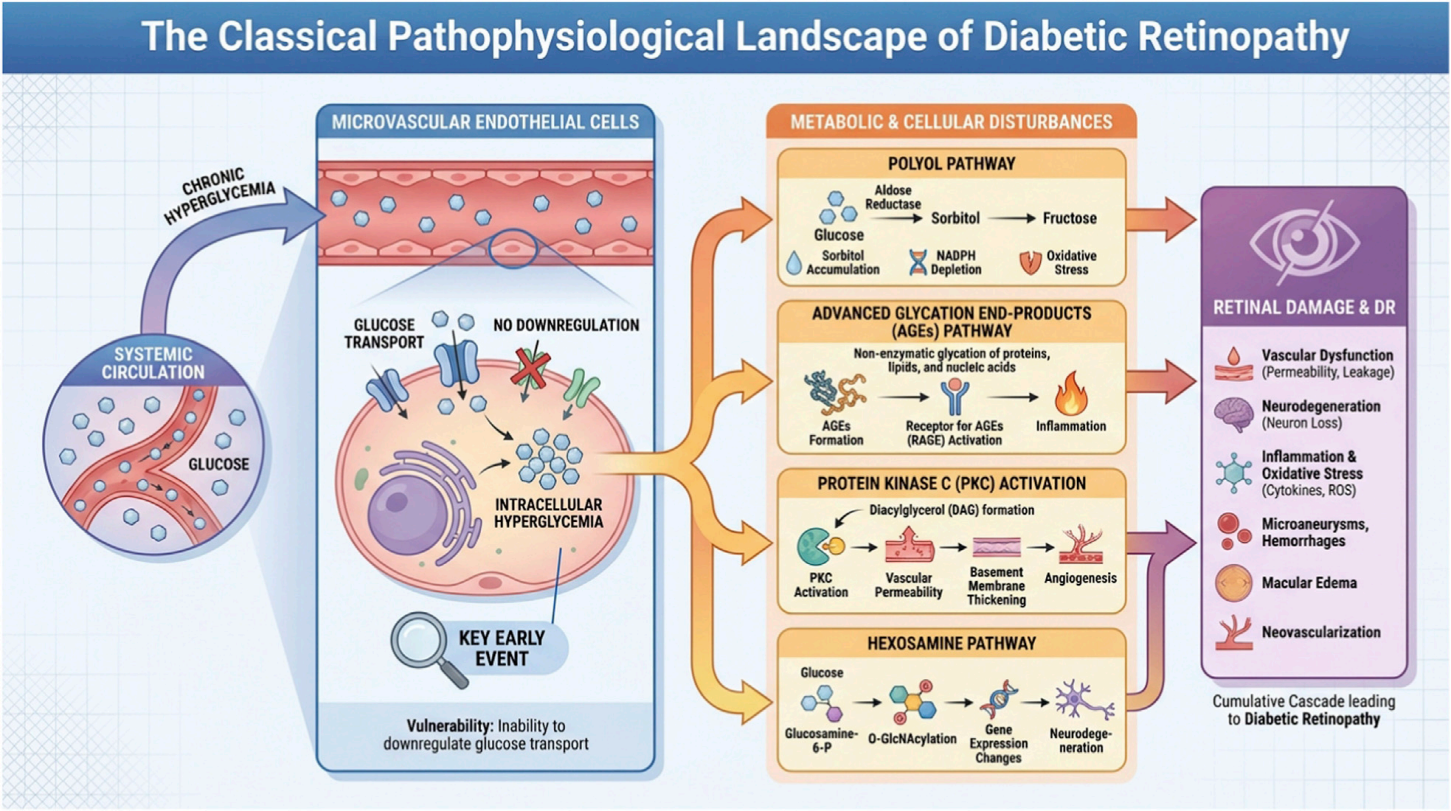
The classical pathophysiological landscape of diabetic retinopathy (DR). Chronic systemic hyperglycemia leads to increased glucose transport into microvascular endothelial cells. A key early event is the inability of these cells to downregulate glucose transport, resulting in intracellular hyperglycemia. This metabolic overload activates four major biochemical pathways: the polyol pathway (leading to sorbitol accumulation and oxidative stress), the advanced glycation end-products (AGEs) pathway (triggering inflammation via RAGE activation), protein kinase C (PKC) activation (causing vascular permeability and angiogenesis), and the hexosamine pathway (altering gene expression). Collectively, these disturbances culminate in retinal damage characterized by vascular dysfunction, neurodegeneration, inflammation, microaneurysms, macular edema, and neovascularization. Figure generated by Gemini Nano Banana Pro.

Chronic hyperglycemia is the initiating factor in diabetic retinopathy (DR), and its pathological effects are primarily mediated through the overactivation of four interconnected classical biochemical pathways: the polyol pathway, the hexosamine pathway, the protein kinase C (PKC) pathway, and the formation of advanced glycation end-products (AGEs) (Hussain et al., 2023; Mahajan et al., 2019) (Table 1; Figure 1, created using Gemini Nano Banana Pro).

**TABLE 1:** Summary of classical pathophysiological pathways in diabetic retinopathy.

Name	Content/Function	Citation (PMID)
Polyol Pathway	Activated by high glucose, causing aldose reductase to convert glucose to sorbitol. This depletes NADPH (impairing antioxidant defenses) and causes osmotic stress. It also links to the AGE pathway.	Hussain et al. (2023), Mahajan et al. (2019), Paul et al. (2020)
Hexosamine Biosynthesis Pathway	Excess fructose-6-phosphate is shunted to produce UDP-N-acetylglucosamine, leading to O-GlcNAcylation of proteins. This PTM alters gene expression, promoting pro-inflammatory and pro-fibrotic factors.	Hussain et al. (2023), Mahajan et al. (2019), Paul et al. (2020)
Protein Kinase C (PKC) Pathway	Hyperglycemia increases diacylglycerol (DAG) synthesis, activating PKC isoforms (especially PKC-β). This alters the expression of vasoactive factors (e.g., VEGF), increasing vascular permeability, inflammation, and apoptosis.	Mahajan et al. (2019), Paul et al. (2020), Kitada et al. (2010)
Advanced Glycation End-products (AGEs) Formation	Non-enzymatic glycation of proteins, lipids, and nucleic acids forms irreversible AGEs. AGEs cross-link extracellular matrix proteins, increasing vascular stiffness, and activate the RAGE receptor, triggering damaging intracellular signaling.	Mahajan et al. (2019), Kitada et al. (2010)

### 2.2 Neurovascular unit damage and clinical pathology

This noxious milieu of oxidative and inflammatory stress directly damages the retinal neurovascular unit (NVU)—an integrated system comprising endothelial cells, pericytes, Müller cells, astrocytes, microglia, and neurons (Lei et al., 2026; Yang H. et al., 2025). Early pathological events include the apoptosis of retinal endothelial cells and pericytes. Pericyte loss (“dropout”), driven by factors such as PKC-δ activation and AGE accumulation, is a hallmark of early DR, leading to capillary destabilization and the initial breakdown of the inner blood-retinal barrier (BRB) (Mahajan et al., 2019; Paul et al., 2020; Kitada et al., 2010). Concurrently, glial cells, particularly Müller cells, become activated (gliosis), exacerbating the inflammatory environment and promoting neuronal dysfunction and pathological neovascularization (Yang H. et al., 2025). Neurodegeneration, especially the loss of retinal ganglion cells, is also recognized as an early component of DR, underscoring its nature as a neurovascular disease (Lei et al., 2026).

The collective dysfunction of the NVU manifests as distinct clinical pathologies: BRB breakdown leads to vascular leakage and diabetic macular edema (DME), a primary cause of vision loss; progressive capillary occlusion causes retinal ischemia, driving significant upregulation of pro-angiogenic factors like VEGF, which in turn fuels pathological neovascularization—the hallmark of proliferative DR (PDR)—predisposing the eye to vitreous hemorrhage and tractional retinal detachment (Mahajan et al., 2019; Paul et al., 2020).

### 2.3 Metabolic memory: the epigenetic basis of persistent damage

“Metabolic memory” describes the phenomenon where the detrimental effects of prior hyperglycemia persist and drive DR progression even after glycemic control is achieved (Natarajan, 2021; Zhang et al., 2012). Clinical evidence from the Diabetes Control and Complications Trial (DCCT) and its follow-up, the Epidemiology of Diabetes Interventions and Complications (EDIC) study, demonstrated that the risk reduction from early intensive glycemic therapy in type 1 diabetes persisted long after glycemic levels converged with those of the conventionally treated group (Natarajan, 2021; Zhang et al., 2012).

**FIGURE 2:**
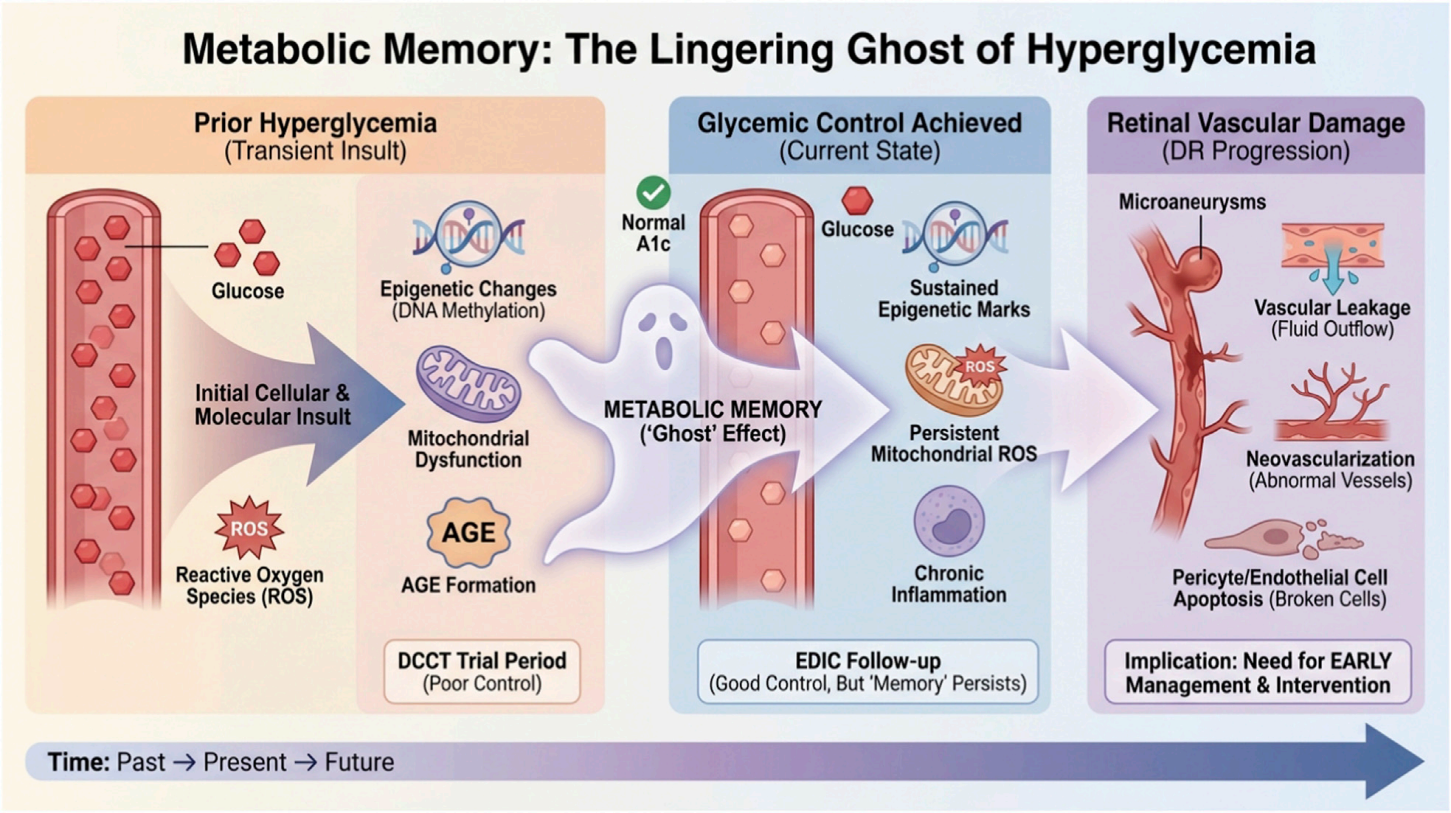
Metabolic memory: The lingering ghost of hyperglycemia. This schematic illustrates the concept of “metabolic memory,” where prior periods of hyperglycemia induce lasting cellular damage that persists even after glycemic control is achieved. During the initial insult, high glucose levels trigger reactive oxygen species (ROS) production, mitochondrial dysfunction, AGE formation, and epigenetic changes (DNA methylation). Even when normal HbA1c levels are restored, these sustained epigenetic marks, persistent mitochondrial ROS, and chronic inflammation continue to drive retinal vascular damage. This progression leads to microaneurysms, vascular leakage, neovascularization, and cell apoptosis, highlighting the critical need for early management and intervention. Figure generated by Gemini Nano Banana Pro.

The core mechanisms involve self-perpetuating biochemical alterations. Transient hyperglycemia triggers the overproduction of ROS and AGEs, causing irreversible protein and lipid modifications and sustaining cellular stress. These insults are maintained through stable epigenetic modifications, such as changes in DNA methylation and histone modifications, which sustain pro-inflammatory and pro-fibrotic gene expression even after glycemic normalization (Natarajan, 2021; Zhang et al., 2012; Kumari et al., 2020). Furthermore, regulation at the epitranscriptomic level, particularly N6-methyladenosine (m6A) RNA methylation and the dysregulation of key enzymes like the demethylase FTO, has been implicated in propagating metabolic dysfunction (Yu et al., 2026) (Figure 2, created using Gemini Nano Banana Pro).

AMP-activated protein kinase (AMPK), a key cellular energy sensor, represents a promising therapeutic target for counteracting metabolic memory. AMPK activation can interrupt hyperglycemic damage by mitigating oxidative stress, suppressing NF-κB-mediated inflammation, and modulating enzymes involved in epigenetic modifications, suggesting its potential to reverse the harmful epigenetic memory of hyperglycemia and halt DR progression (Khan et al., 2026).

## 3 m6A modification in diabetic retinopathy

N6-methyladenosine (m6A), the most abundant mRNA modification, is dynamically regulated by “writer” (e.g., METTL3/METTL14), “eraser” (FTO, ALKBH5), and “reader” (e.g., YTHDF family, IGF2BP family) proteins (Wang et al., 2022; Zhuo et al., 2022; Wang et al., 2021). This system mediates rapid responses to stress, partly via “m6A switches” that alter RNA structure (Zhang, 2017). Readers have distinct functions: YTHDF1/YTHDF3 facilitate translation (Wang et al., 2021), while YTHDF2 promotes mRNA degradation (Wang et al., 2025b) Dysregulated m6A is implicated in diabetes and its complications (Tang et al., 2025; Wang et al., 2020) (Table 2).

**TABLE 2:** Key m6A regulators and their roles in diabetes and related pathologies.

Name	Content/Function	Citation (PMID)
METTL3/METTL14	**Writer Complex**: The primary m6A methyltransferase complex. Recruited to DNA damage sites to deposit m6A on RNA, facilitating rapid repair.	(Wang et al., 2022; Zhang, 2017)
METTL3	**Writer**: Promotes angiogenesis in retinal pathology by modifying *EGR1* mRNA. Stabilizes glycolytic gene transcripts (*SLC2A1*, *HK2*) with IGF2BP2. In diabetic kidney disease, mediates *TfR1* mRNA stabilization, leading to ferroptosis.	(Tao et al., 2025; Zhang et al., 2025b; Huo et al., 2021)
METTL14	**Writer**: Essential for photoreceptor health; loss leads to retinal degeneration. Downregulated in diabetic cardiomyopathy, causing accumulation of lncRNA *TINCR* and promoting pyroptosis.	(Meng et al., 2022; Yang et al., 2022)
WTAP	**Writer**: Upregulated by high glucose in impaired wound healing, leading to endothelial dysfunction.	(Pen et al., 2025; Xiao et al., 2025)
FTO	**Eraser**: Demethylase linked to metabolic control and diabetes risk. Upregulated by high glucose in β-cells. Downregulation in some pathologies increases m6A on angiogenesis/immune transcripts. Pharmacological inhibition can rescue insulin secretion.	(Bornaque et al., 2022; Ma et al., 2024; Ju et al., 2021; Qiu et al., 2021)
ALKBH5	**Eraser**: Demethylase. Upregulated in neovascular AMD, where it stabilizes *ID2* mRNA to promote angiogenesis. Dysregulation is linked to tau hyperphosphorylation in diabetic cognitive dysfunction.	(Bornaque et al., 2022; Wei et al., 2026; Qu et al., 2023)
YTHDF1/YTHDF3	**Reader**: Facilitate mRNA translation. YTHDF3, with METTL3, stabilizes *TfR1* mRNA in diabetic kidney disease.	(Zhang et al., 2025b; Wang et al., 2021)
YTHDF2	**Reader**: Promotes mRNA degradation. Shapes inflammation by targeting transcripts like *Stat1*.	(Wang et al., 2021; Wang et al., 2025a)
IGF2BP2	**Reader**: In retinal pathology, stabilizes glycolytic gene transcripts with METTL3. In diabetic macular edema (DME), mediates upregulation of SMURF1, disrupting RPE tight junctions.	(Huo et al., 2021; Liang et al., 2025)

**FIGURE 3:**
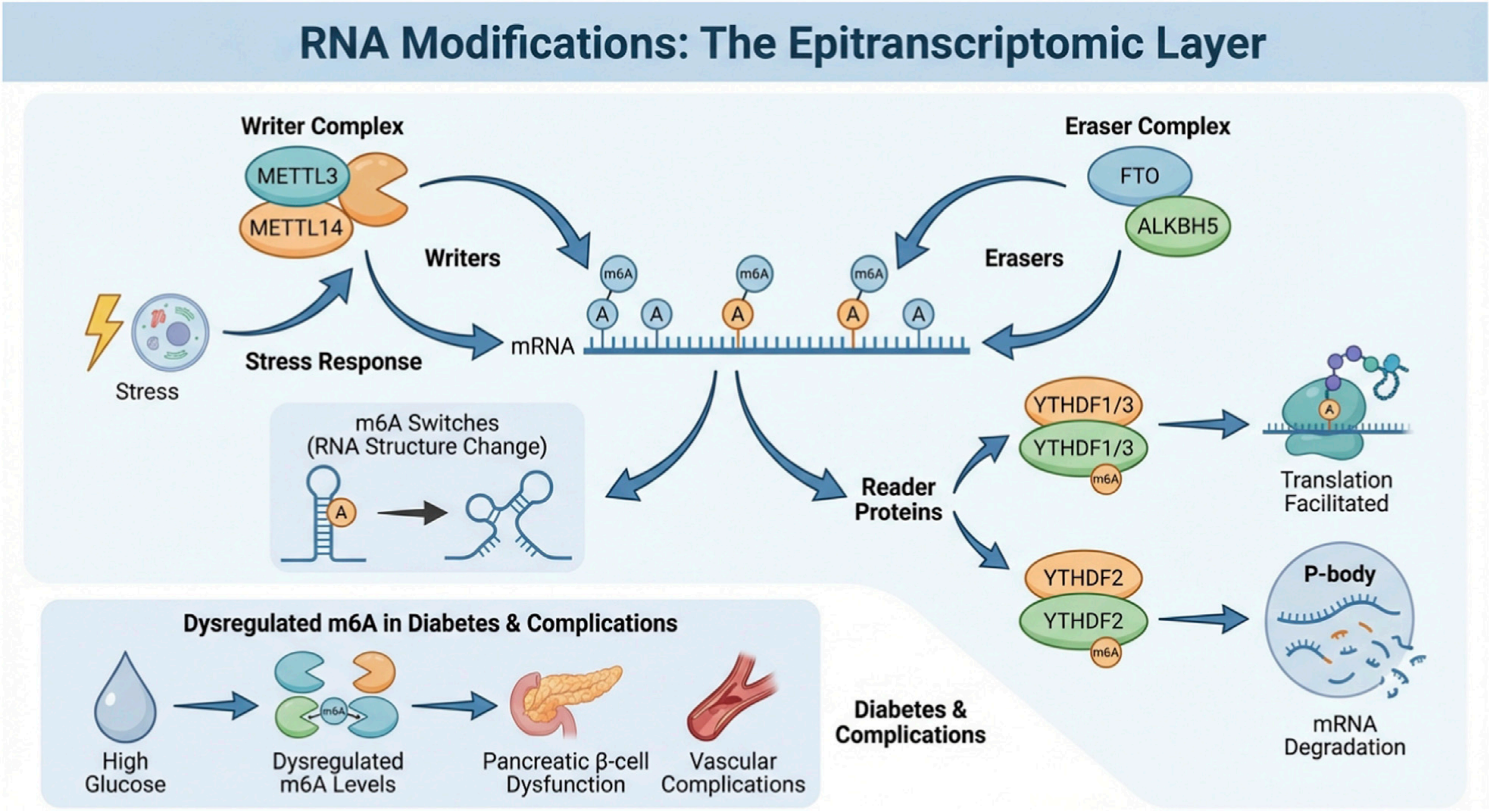
RNA modifications: The epitranscriptomic layer. This diagram depicts the dynamic regulation of N6-methyladenosine (m6A) mRNA methylation, a key epitranscriptomic modification. The process is regulated by “writer” complexes (METTL3/METTL14) that add methyl groups, “eraser” complexes (FTO, ALKBH5) that remove them, and “reader” proteins (YTHDF1/3, YTHDF2) that interpret the marks to facilitate translation or mRNA degradation. m6A modifications can also induce structural changes in RNA (“m6A switches”). In the context of diabetes, high glucose levels lead to dysregulated m6A levels, contributing to pancreatic β-cell dysfunction and vascular complications. Figure generated by Gemini Nano Banana Pro.

High glucose alters m6A machinery; in pancreatic β-cells, it upregulates demethylases *FTO* and *ALKBH5*, decreasing global m6A (Bornaque et al., 2022). High-fat diets can also alter m6A landscapes via the gut microbiota (Liu Y. et al., 2025). In retinal pathology, METTL3 promotes angiogenesis by modifying *EGR1* mRNA (Tao et al., 2025) and, with reader IGF2BP2, stabilizes glycolytic gene transcripts (*SLC2A1*, *HK2*) (Huo et al., 2021). The writer METTL14 is essential for photoreceptor health; its loss causes progressive retinal degeneration due to failed protein trafficking to the outer segment, as m6A is required for expression of phototransduction and ciliogenesis genes (Yang et al., 2022). In a novel DNA repair pathway, the METTL3/METTL14 complex is recruited to DNA damage sites to deposit m6A on local RNAs, facilitating rapid repair (Zhang, 2017) (Figure 3, created using Gemini Nano Banana Pro).

Erasers and readers are also critical. The reader YTHDF2 typically promotes mRNA decay (Wang et al., 2021; Wang et al., 2025c). In neovascular AMD, the demethylase ALKBH5 is upregulated and stabilizes *ID2* mRNA by removing m6A marks, promoting angiogenesis (Wei et al., 2026). In diabetic cognitive dysfunction, ALKBH5 dysregulation is linked to tau hyperphosphorylation (Qu et al., 2023), relevant to DR’s neurodegenerative component (Wang et al., 2025a). The eraser FTO is linked to metabolic control, inflammation, and diabetes risk (Ma et al., 2024; Qiu et al., 2021; Qing et al., 2021). In some pathologies, FTO is downregulated while writers are upregulated, increasing m6A on angiogenesis (*VEGFR*, *MMP9*) and immune (*HLA-G*) transcripts (Qiu et al., 2021). Pharmacological FTO inhibition can rescue insulin secretion in islets (Bornaque et al., 2022). Expression of m6A regulators is controlled by DR-relevant stimuli like NF-κB and hypoxia (Wang et al., 2025b), linking inflammation and ischemia to epitranscriptomic control (Abdolmaleky et al., 2025; Yan et al., 2024). Readers like YTHDF2 also shape inflammation by targeting transcripts like *Stat1* (Wang et al., 2025c).

Downstream, in diabetic macular edema (DME), the reader IGF2BP2 mediates m6A-dependent upregulation of SMURF1, disrupting RPE tight junctions (Liang et al., 2025). Reader expression can also be altered (Qiu et al., 2021). Shared m6A-driven pathways exist across diabetic complications. In diabetic kidney disease, METTL3/YTHDF3-mediated stabilization of *TfR1* mRNA leads to iron accumulation and ferroptosis (Zhang J. et al., 2025; Zhang et al., 2025c; Wu H. et al., 2025; Lu et al., 2021). In diabetic cardiomyopathy, METTL14 is downregulated, leading to accumulation of the lncRNA *TINCR*, which stabilizes *NLRP3* mRNA and promotes pyroptosis (Meng et al., 2022). In another cardiomyopathy model, FTO downregulation increased global m6A; restoring FTO improved cardiac function (Ju et al., 2021). In impaired wound healing, the writer WTAP is upregulated by high glucose, causing endothelial dysfunction (Pen et al., 2025; Xiao et al., 2025).

## 4 Gut microbiota: a systemic driver of diabetic retinopathy

The pathogenesis of diabetic retinopathy (DR) extends far beyond the local retinal environment, involving a complex network of systemic metabolic dysregulation, inflammation, and inter-organ crosstalk. The gut microbiome (including bacterial, fungal, and viral components) serves as a critical regulator, profoundly influencing the initiation and progression of DR through the “gut-retina axis” (Huang et al., 2021; Lin et al., 2025; Zhang et al., 2025a).

### 4.1 The gut microbiome: from healthy baseline to dysbiosis

#### 4.1.1 The healthy gut microbiome: a reference baseline

A healthy gut microbiome is characterized by high taxonomic diversity, functional redundancy, and stability. At the phylum level, a predominance of Firmicutes and Bacteroidetes (comprising >90% of the community), with lesser contributions from Actinobacteria, Proteobacteria, and Verrucomicrobia, is considered a hallmark of eubiosis (Badal et al., 2020). At the genus level, key health-associated members include butyrate-producing genera (Faecalibacterium prausnitzii, Roseburia, Eubacterium), anti-inflammatory taxa (Akkermansia muciniphila, Bifidobacterium), and fiber-degrading specialists (Prevotella, Ruminococcus) (Badal et al., 2020; Qin et al., 2010). Functionally, a healthy microbiome is characterized by robust production of SCFAs (particularly butyrate, providing ∼5–10% of daily caloric intake via colonocytes), balanced bile acid metabolism, synthesis of vitamins (K, B12, folate), and maintenance of intestinal barrier integrity through competitive exclusion of pathobionts and reinforcement of tight junction proteins (Badal et al., 2020). The concept of a “core microbiome” coexists with substantial inter-individual variability influenced by diet, genetics, age, and geography. Establishing this baseline is essential for evaluating the degree and pathological significance of dysbiosis in DR (Badal et al., 2020; Qin et al., 2010).

#### 4.1.2 Gut microbiota alterations in diabetes and DR

While gut microbiota alterations in diabetes mellitus and GDM are well-characterized, a distinct gut microbial signature specifically associated with DR has emerged from recent clinical studies, particularly from Asian cohorts. Huang et al. (2021) demonstrated that Chinese DR patients exhibit significantly reduced gut microbial alpha diversity, with consistent depletion of SCFA-producing genera (Faecalibacterium, Eubacterium_hallii_group, Roseburia) and enrichment of Gram-negative *Bacteroides* (Huang et al., 2021). This depletion of SCFA producers appears to be a distinguishing feature of DR relative to other diabetic complications. Stratification analysis based on a Bacteroidetes/Firmicutes ratio ≥1.05 revealed distinct profiles within DR: patients with proliferative DR (PDR) show reductions in both species richness and evenness, while those with clinically significant macular edema (CSME) primarily exhibit reduced richness (Khan et al., 2024). Indian cohort studies further demonstrate that a markedly elevated Firmicutes/Bacteroidetes ratio correlates with progression from non-proliferative to proliferative DR, suggesting the ratio may serve as a stage-specific biomarker. Comparative analysis reveals that the DR gut microbiome shares features with DKD (e.g., depletion of Bifidobacterium) but also exhibits unique characteristics, including a more pronounced loss of butyrate producers and enrichment of pro-inflammatory taxa (e.g., *Bacteroides* ovatus, *Escherichia coli*). In contrast, the gut dysbiosis of diabetic neuropathy is characterized by an altered Bacillota/Bacteroidota ratio with increased *Lactobacillus* abundance (Jia et al., 2025). These findings collectively establish the “DR gut” as a distinct microbial entity, positioning gut microbiome profiling as a promising non-invasive tool for DR risk stratification and early detection.

Patients with gestational diabetes mellitus (GDM) exhibit decreased Firmicutes, increased Bacteroidetes, along with elevated Desulfobacterota and *Fusobacterium* (Zhang et al., 2020). In adult type 2 diabetes (T2D) cohorts, the Firmicutes/Bacteroidetes ratio differs significantly from controls (Wu IW. et al., 2025). Notably, patients with type 1 diabetes (with or without comorbid depression) display higher gut microbial alpha diversity compared to healthy controls, with a higher abundance of Megasphaera specifically linked to depression (Petrak et al., 2022). In a non-pathogenic chronic viral infection model, SIV-infected vervet monkeys also exhibit increased microbial diversity during the chronic phase, suggesting that increased diversity is not universally pathological (Jasinska et al., 2020). Dysbiosis is exacerbated under acute systemic stress; for instance, in T2D patients with concurrent COVID-19 infection, beneficial bacteria (Bifidobacterium, *Lactobacillus*) decrease while opportunistic fungi (*Candida* spp.) increase (Petakh et al., 2023). High-fat diet (HFD) rapidly induces dysbiosis, with even short-term (4-day) feeding increasing the Firmicutes/Bacteroidetes ratio and enriching pro-inflammatory families (Ruminococcaceae, Lachnospiraceae) (Liu Y. et al., 2025; Las Heras et al., 2019). GDM-associated dysbiosis exhibits heterogeneity based on fetal birth weight outcomes: the macrosomia group shows further decreased Firmicutes and increased Bacteroidetes, while the fetal growth restriction group displays opposite patterns (Zhang et al., 2020). GDM patients unresponsive to lifestyle interventions show significant alterations in beneficial genera (Blautia, Faecalibacterium) (Ye et al., 2019).

### 4.2 Role of gut microbiota in diabetic complications

Dysbiosis is closely linked to multiple diabetic complications. In diabetic kidney disease (DKD), patients exhibit reduced beneficial bacteria (Bifidobacterium, Enterobacterium), correlating with central obesity, hypertension, and chronic inflammatory markers (Dai M. et al., 2025). Mendelian randomization studies confirm Bacteroidetes (OR 1.58) and Akkermansia (OR 1.46) as risk factors for DN, while Coprococcus 2 (OR 0.68) plays a protective role (Han et al., 2024). Machine learning analyses further identify decreased Gemmiger as a key biomarker for DKD (Wu IW. et al., 2025). In painful diabetic neuropathy models, dysbiosis is characterized by an altered Bacillota/Bacteroidota ratio and increased *Lactobacillus* abundance, correlating with elevated pro-inflammatory cytokines in the spinal cord (Jia et al., 2025). The comorbidity of gut dysbiosis with cognitive dysfunction has also been demonstrated, with specific amplicon sequence variants from Muribaculaceae and Ruminococcus correlating with disease phenotypes (Yang J. et al., 2025). Notably, while DKD and DR share some common dysbiosis features (e.g., depletion of Bifidobacterium), DR is distinguished by a more pronounced depletion of SCFA-producing genera and enrichment of pro-inflammatory Gram-negative bacteria, suggesting that the “DR gut” possesses a unique microbial signature that could serve as a basis for developing disease-specific biomarkers and therapeutic strategies (Sadeghi et al., 2026).

### 4.3 Intestinal barrier function and host regulation

The host actively shapes the gut microbiota through multiple mechanisms. Intestinal epithelial cells (such as tuft cells and Paneth cells) secrete antimicrobial proteins (e.g., SPRR2A) that selectively kill Gram-positive bacteria (Hu et al., 2021). Goblet cells can form goblet cell-associated antigen passages (GAPs) that actively transport luminal contents to lamina propria antigen-presenting cells, with antibiotic-induced dysbiosis increasing GAP formation and promoting bacterial translocation (Knoop et al., 2017).

Intestinal barrier integrity is modulated by bile acids: deoxycholic acid (DCA) disrupts the barrier, while lithocholic acid (LCA) and tauroursodeoxycholic acid (TUDCA) exert protective effects (Shi et al., 2023). Host genetic factors also contribute; for example, Tcf4, the protein product of the T2D susceptibility gene TCF7L2, controls Paneth cell differentiation, and its loss leads to reduced antimicrobial α-defensins and disrupted microbial balance (Janeckova et al., 2025).

#### 4.3.1 Short-chain fatty acids (SCFAs)

Gut microbiota influences host pathophysiology extensively through its metabolites. Short-chain fatty acids (SCFAs) such as butyrate can cross the blood-eye barrier and suppress intraocular inflammation (Chen N. et al., 2021); they also promote GLP-1 secretion by activating GPR41/GPR43, improving insulin sensitivity (Mei et al., 2025). Critically, butyrate enters the TCA cycle to produce α-ketoglutarate (α-KG), a necessary cofactor for m6A demethylases (FTO, ALKBH5), thereby directly promoting RNA demethylation and linking SCFA availability to epitranscriptomic regulation (Zhang et al., 2025a).

However, SCFA effects are context-dependent, as excess acetate can activate the renin-angiotensin system to induce early kidney injury (Yu et al., 2024). The HDAC inhibitory activity of butyrate also represents a key epigenetic mechanism: by inhibiting histone deacetylases (HDACs), butyrate globally alters chromatin accessibility, which can indirectly affect the transcription of m6A writers and erasers. This dual mechanism—direct α-KG-mediated demethylase modulation and indirect HDAC-mediated transcriptional regulation—positions SCFAs at the nexus of the gut-microbiota-m6A axis.

#### 4.3.2 Secondary bile acids: FXR/TGR5 signaling

Secondary bile acids, produced by the gut microbiota through deconjugation and dehydroxylation of primary bile acids, represent another class of signaling molecules with significant epigenetic implications. Deoxycholic acid (DCA) and lithocholic acid (LCA) activate the nuclear farnesoid X receptor (FXR) and the membrane-bound Takeda G protein-coupled receptor 5 (TGR5), which modulate histone acetylation and methylation status in intestinal and distant tissues (Wang ZL. et al., 2025; Li and Chiang, 2014). FXR activation has been shown to regulate the expression of m6A writers in hepatocytes, providing a direct link between bile acid signaling and epitranscriptomic control. In the context of DR, FXR/TGR5 agonists have demonstrated anti-inflammatory and anti-angiogenic effects in retinal endothelial cells, suggesting that bile acid signaling may represent a novel therapeutic axis (Wang ZL. et al., 2025).

#### 4.3.3 Trimethylamine N-Oxide (TMAO)

Trimethylamine N-oxide (TMAO) is another key metabolite, with elevated levels in DR patients positively correlating with disease severity (Zhou et al., 2022). TMAO promotes kidney injury by activating the NLRP3 inflammasome and NF-κB pathways, and can downregulate vitamin D receptor expression (Liu J. et al., 2025; Zheng X. et al., 2024). Given that NF-κB signaling directly regulates the expression of m6A writers such as METTL3, TMAO may influence the m6A epitranscriptome through NF-κB-dependent transcriptional reprogramming. Recent evidence demonstrates that TMAO activates the NF-κB-METTL3 signaling axis, thereby upregulating METTL3 expression and altering m6A modification patterns on inflammatory gene transcripts, including IL-6 and TNF-α (Chen J. et al., 2025). This finding provides a direct mechanistic link between a gut microbial metabolite and the m6A machinery, reinforcing the concept of a microbiota–metabolite–m6A regulatory cascade in DR.

#### 4.3.4 Branched-chain amino acids (BCAAs)

Dysregulated branched-chain amino acid (BCAA) metabolism leads to elevated circulating BCAAs, inducing mitochondrial dysfunction via mTOR signaling (Zheng H. et al., 2024). In a diabetic cardiomyopathy model, reduced microbial BCAA degradation capacity decreases FGF21, leading to myocardial BCAA accumulation and dysfunction (Zheng H. et al., 2024).

#### 4.3.5 Tryptophan derivatives: AhR signaling

Indole derivatives from tryptophan metabolism, such as indole-3-propionic acid (IPA) and indole-3-aldehyde (IAld), activate the aryl hydrocarbon receptor (AhR), a ligand-activated transcription factor that integrates microbial signals with host gene expression (Hubbard et al., 2015). AhR activation regulates the expression of m6A writers (METTL3) and erasers (ALKBH5) through transcriptional and post-transcriptional mechanisms. IPA, in particular, has been shown to protect retinal pigment epithelium from oxidative stress through Nrf2 activation, linking tryptophan metabolism to retinal protection (Hubbard et al., 2015).

#### 4.3.6 Homogentisic acid (HGA) and other metabolites

Homogentisic acid (HGA), a newly identified pathogenic metabolite, increases with HFD and promotes metabolic dysfunction by increasing m6A modification of Ehmt2 mRNA, reducing EHMT2 protein expression, and activating transposable elements (Liu Y. et al., 2025). Conversely, beneficial metabolites such as 2-oxoindole-3-acetate (OAA) produced by Romboutsia ilealis alleviate obesity by promoting YTHDF2 degradation (Zhu et al., 2026).

### 4.4 Gut-retina axis and retinal pathology

The association between gut dysbiosis and ocular diseases has been conceptualized as the “gut-retina axis” (Kaur et al., 2025; Rowan and Taylor, 2018). A high-glycemic diet induces AMD-like retinal pathology associated with Firmicutes (particularly Clostridia) expansion, while a low-glycemic diet is protective and linked to higher Bacteroidetes (Rowan and Taylor, 2018). Studies in germ-free mice demonstrate that HFD can directly alter the RPE/choroid transcriptome independently of the microbiota, upregulating genes involved in angiogenesis, inflammation, and immune responses (Xiao et al., 2022). Clinical studies confirm that DR patients exhibit significantly reduced gut microbial alpha diversity, with depletion of beneficial genera (Faecalibacterium, Eubacterium_hallii_group) and enrichment of *Bacteroides* (Huang et al., 2021). Stratification analysis based on a Bacteroidetes/Firmicutes ratio ≥1.05 reveals that proliferative DR patients show reductions in both species richness and evenness, while clinically significant macular edema primarily exhibits reduced richness (Khan et al., 2024).

### 4.5 Interplay between gut microbiota and host epigenetic/epitranscriptomic modifications

Gut microbiota influences host epigenetic modifications through multiple pathways. Folate produced by beneficial bacteria contributes to S-adenosylmethionine (SAM) synthesis via one-carbon metabolism, providing substrates for DNA, histone, and RNA methylation (Zhang et al., 2025a). In colorectal cancer, tumor-enriched bacteria (*Fusobacterium* nucleatum, Hungatella hathewayi) directly upregulate host DNA methyltransferases, leading to promoter hypermethylation of tumor suppressor genes (Park et al., 2024).

Bidirectional interactions exist between m6A modification and gut microbiota. HFD-induced dysbiosis directly increases m6A levels in epididymal white adipose tissue (Liu Y. et al., 2025). The SCFA butyrate enters the TCA cycle to produce α-ketoglutarate (α-KG), a necessary cofactor for m6A demethylases, thereby promoting RNA demethylation (Zhang et al., 2025a). Conversely, host m6A machinery shapes the microbiota: intestinal upregulation of FTO ameliorates arsenic-induced neurotoxicity by restoring the abundance of the beneficial, hydrogen sulfide-producing bacterium Desulfovibrio fairfieldensis (Chen R. et al., 2025).

In diabetic complications, m6A modifications are widely involved in pathological processes. In DKD, METTL3 is upregulated in podocytes and activates Notch signaling by stabilizing MDM2 mRNA (Wu H. et al., 2025), while METTL14 promotes podocyte injury by facilitating SIRT1 mRNA degradation (Lu et al., 2021). In the intestine, METTL14 is essential for maintaining epithelial homeostasis, and its loss leads to mucosal barrier dysfunction (Wang H. et al., 2025). The m6A readers IGF2BP1 and YTHDC1 also protect intestinal barrier integrity by regulating the tight junction protein occludin (Wang H. et al., 2025).

## 5 Translational horizons: biomarkers and therapeutic strategies

**FIGURE 4:**
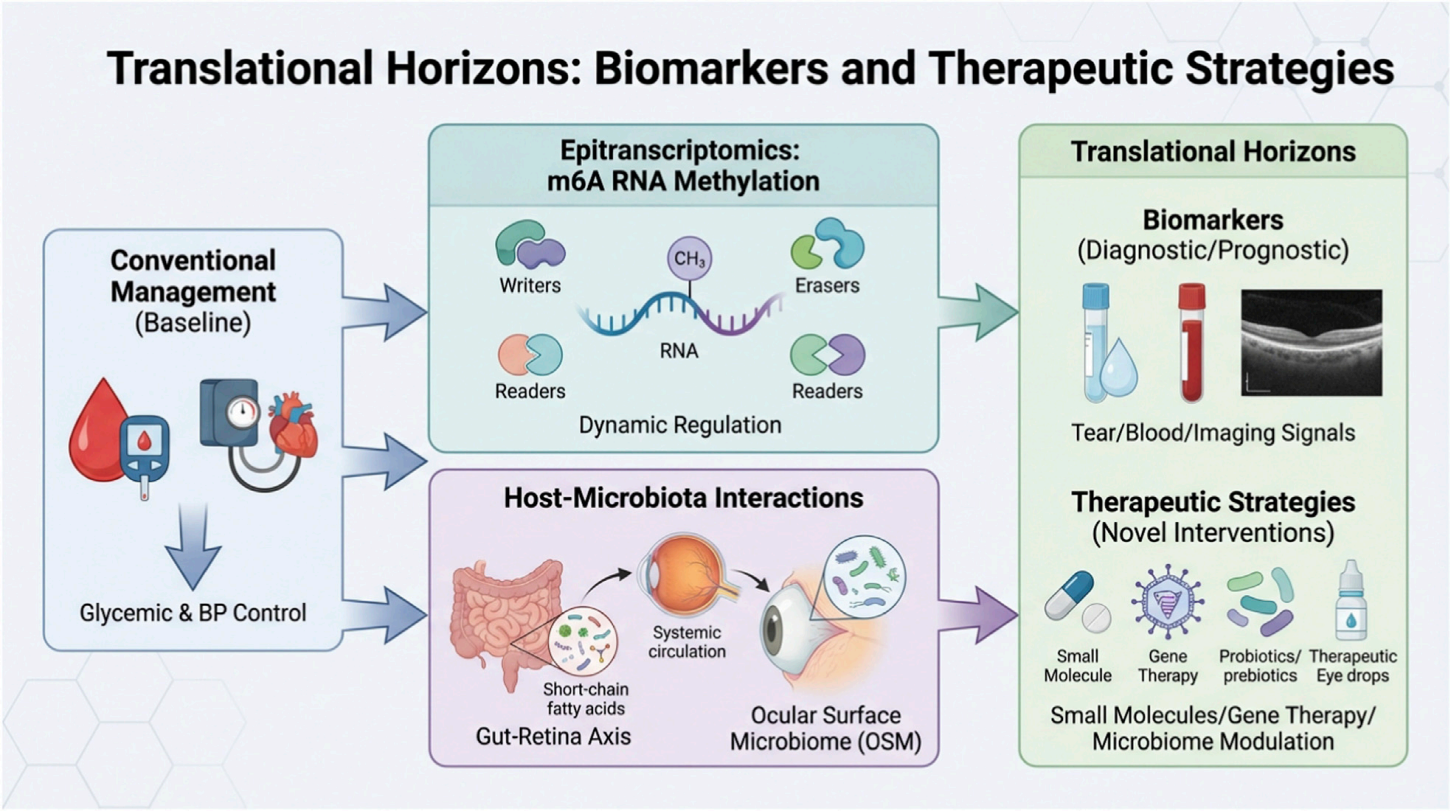
Translational horizons: Biomarkers and therapeutic strategies. This figure outlines the shift from conventional management to novel translational approaches for DR. While conventional management focuses on glycemic and blood pressure control, emerging research targets epitranscriptomics (specifically m6A RNA methylation dynamics) and host-microbiota interactions (including the gut-retina axis and ocular surface microbiome). These new frontiers offer potential for advanced biomarkers (derived from tears, blood, or imaging signals) and novel therapeutic strategies, such as small molecule inhibitors, gene therapy, probiotics/prebiotics, and specialized therapeutic eye drops. Figure generated by Gemini Nano Banana Pro.

An in-depth understanding of the molecular mechanisms underlying diabetic retinopathy (DR) has opened new avenues for clinical translation (Figure 4, created using Gemini Nano Banana Pro). Epitranscriptomics (particularly m6A RNA methylation) and host-microbiota interactions (including the gut-retina axis and the ocular surface microbiome) represent two particularly promising frontiers. The synthesis of these mechanistic insights provides a rich resource for the development of innovative biomarkers (Chaitankar et al., 2016).

### 5.1 Epitranscriptomic and genetic markers

m6A RNA methylation, regulated by “writers” (METTL3, METTL16), “erasers” (FTO, ALKBH5), and “readers” (YTHDF proteins), serves as an ideal sensor of the diabetic metabolic state (Su et al., 2022; Xu et al., 2021). Hyperglycemia can directly induce alterations in the m6A machinery; for example, high glucose increases WTAP expression, affecting the stability of metabolic enzyme mRNAs such as *HK2* (Niu et al., 2025). The principle that the m6A landscape serves as a sensitive barometer of systemic disease is supported by findings in COVID-19 (for which diabetes is a major risk factor), where infection triggers widespread changes in m6A regulators, including upregulation of WTAP, RBM15, and YTHDC1, and downregulation of IGF2BP1 (Qian et al., 2023). The m6A reader IGF2BP2 promotes diabetic macular edema by recognizing and stabilizing m6A-modified SMURF1 mRNA, making SMURF1 itself a potential biomarker (Liang et al., 2025). It is important to clarify that the lens m6A alterations observed in diabetic cataract patients, while epitranscriptomic in nature, reflect the metabolic consequences of hyperglycemia on lens epithelial cells rather than contributing directly to DR pathogenesis. These findings are therefore not discussed in detail here, as they pertain to a distinct diabetic ocular complication.

Systems-level network analyses are being employed to map the broader regulatory landscape controlled by the m6A machinery in T2DM. Co-expression network analysis of human islet β-cell transcriptomes identified 985 genes co-expressed with m6A regulators (Co-m6ARs), which were significantly enriched in key T2DM-related pathways such as metabolic processes and MAPK signaling. This analysis pinpointed a set of high-confidence biomarker candidates, including *CCNL2*, *CSAD*, *COX5A*, and *GAB2*, with *GAB2* harboring expression quantitative trait loci (eQTLs) strongly associated with diabetes (Chaitankar et al., 2016; Chen C. et al., 2021).

Clinical studies have confirmed that peripheral blood from T2DM patients exhibits decreased total m6A levels, with altered expression of key enzymes. The eraser FTO is upregulated in peripheral blood (correlating with disease severity), while the writer METTL3 is downregulated, and low levels of the reader IGF2BP3 are associated with an increased risk of developing T2DM (Hlavackova et al., 2025). Diabetic wound tissues show characteristic upregulation of ALKBH5 alongside downregulation of METTL3, METTL14, and WTAP (Zhang et al., 2024).

### 5.2 Microbiome-based markers

The direct link between gut dysbiosis and diabetic complications such as DKD provides a framework for developing microbiome-based biomarkers for DR. Patients with DKD exhibit reduced abundance of beneficial bacteria (Bifidobacterium, Enterobacterium), correlating with central obesity, hypertension, and chronic inflammatory markers (Dai M. et al., 2025). Machine learning models have successfully utilized gut microbiota profiles to predict DKD status (Wu IW. et al., 2025). DR patients show significantly reduced gut microbial alpha diversity, with depletion of beneficial genera (Faecalibacterium, Eubacterium_hallii_group) and enrichment of *Bacteroides* (Huang et al., 2021). Stratification analysis based on a Bacteroidetes/Firmicutes ratio ≥1.05 reveals that patients with proliferative DR (PDR) exhibit reductions in both species richness and evenness, while those with clinically significant macular edema primarily show reduced richness (Khan et al., 2024).

The gut mycobiome is also emerging as a key source of biomarkers. Patients with T2DM and diabetic foot (DF) exhibit distinct gut mycobiome dysbiosis, with Rhodotorula mucilaginosa significantly decreased in T2DM, while *Candida* species are increased exclusively in the T2DM-DF group, suggesting their potential as biomarkers for severe complications (Cai et al., 2024).

The ocular surface microbiome (OSM) has emerged as a potential non-invasive source of biomarkers for diabetic complications. Diabetic patients with dry eye show reduced microbial diversity on the conjunctival surface, with an increase in pathogenic bacteria (*E. coli*, *Chlamydia trachomatis*) (Chen et al., 2023a). The OSM provides a unique window into systemic diabetic status: the tear film metabolome reflects both local microbial activity and circulating metabolite levels (including glucose, SCFAs, and cytokines), and OSM dysbiosis correlates with diabetes duration and ocular surface disease severity. While the OSM does not directly parallel the gut-retina axis in terms of disease mechanism, its composition and metabolic output serve as a non-invasive, easily accessible biomarker panel that may complement gut microbiome analysis in a multi-site microbiome-based diagnostic framework for DR. In infectious keratitis, a random forest machine learning model based on OSM profiles can differentiate between types of microbial keratitis with high accuracy (96.25%) (Ren et al., 2022). However, the choice of sampling method (tear strips vs. conjunctival swabs) and downstream processing (DNA extraction kits, bioinformatics pipelines) can significantly influence results, making standardization of the workflow crucial for robust biomarker development (Chen et al., 2023b; Herzog et al., 2023).

### 5.3 Therapeutic strategies

#### 5.3.1 Targeting m6A machinery

Given the widespread dysregulation of m6A modifications in DR and diabetic complications, m6A-modifying enzymes represent potential therapeutic targets. In a GDM model, lentiviral-mediated restoration of FTO expression rescued hypertrophic and senescent cardiac phenotypes in offspring (Yu et al., 2026). In diabetic kidney disease, podocyte-specific knockout of Mettl3 ameliorated albuminuria and renal pathology (Wu H. et al., 2025). However, due to the tissue-specificity and functional diversity of the m6A machinery, careful development of tissue-specific intervention strategies is required.

#### 5.3.2 Targeting gut microbiota

Probiotics, prebiotics, and fecal microbiota transplantation (FMT) have shown therapeutic potential in animal models. Bifidobacterium animalis subsp. lactis BB-12 alleviates DR by modulating the gut microbiota (Lin et al., 2025). Myricetin increases the abundance of beneficial bacteria (Roseburia, Faecalibaculum, Bifidobacterium), restores intestinal barrier integrity, and alleviates diabetic cardiomyopathy (Zhu et al., 2024). Exercise improves endothelial progenitor cell function in diabetic mice through FMT, enriching Akkermansia and increasing plasma GLP-1 (Dai X. et al., 2025). A probiotic therapy designed to restore ethanolamine-metabolizing function successfully reversed gut permeability and improved glucose metabolism in preclinical models (Mishra et al., 2023).

#### 5.3.3 Combined intervention strategies

Given the bidirectional interaction between the gut microbiota and m6A modifications, combined intervention strategies (e.g., probiotics combined with m6A enzyme modulators) may produce synergistic effects. By remodeling the microbiota-metabolite-m6A axis, it may be possible to more effectively reverse the pathological progression of DR.

## 6 Conclusion and future perspectives

The pathogenesis paradigm of diabetic retinopathy has evolved from a singular glucose-centric model to a multifactorial model encompassing metabolic memory, epigenetic (transcriptomic) reprogramming, and crosstalk between the gut microbiota. The **bidirectional interaction** between the **gut microbiota** and **RNA m6A modification** is a central link in this new paradigm. Gut dysbiosis participates in solidifying the hyperglycemia-induced pathological state by influencing the host’s (including retinal cells) m6A epitranscriptome; conversely, dysregulation of the host’s m6A machinery reacts back on the gut environment, forming a feedback loop that drives disease progression.

We acknowledge that the evidence establishing direct causal links within the microbiota-m6A-DR axis remains preliminary. Current studies are largely associative, and mechanistic validation in retinal-specific contexts—particularly through interventional studies combining gnotobiotic models with epitranscriptomic profiling—is urgently needed. The field faces significant challenges, including the tissue-specific complexity of m6A regulation, the inter-individual variability of the gut microbiome, and the lack of standardized multi-omics integration frameworks. Despite these limitations, the accumulating evidence strongly supports the microbiota-m6A crosstalk as a promising framework for understanding DR pathogenesis.

Future research needs to utilize multi-omics technologies (metagenomics, epitranscriptomics, metabolomics) in longitudinal clinical cohorts and more refined animal models to further map the causal relationships within the “microbiota-metabolite-m6A-retinal pathology” axis. Specific priorities include: (1) longitudinal multi-omics profiling of DR patients to establish temporal causality; (2) interventional studies combining FMT with m6A modulator treatment in animal models to demonstrate therapeutic synergy; (3) development of tissue-specific m6A-targeting approaches for retinal applications; and (4) validation of combined gut microbiome and circulating m6A biomarkers in diverse clinical cohorts. Furthermore, the FXR/TGR5 signaling axis represents a particularly promising translational opportunity: FXR agonists, already in clinical development for metabolic liver disease, could be repurposed for DR by leveraging their dual anti-inflammatory and epitranscriptomic regulatory effects, making them attractive candidates for future preclinical evaluation. The ultimate goal is to translate these findings into clinical practice: early risk stratification through the detection of integrated biomarkers and the implementation of precision therapies based on the microbiome and epitranscriptome, thereby more effectively preventing and reversing this blinding complication.
